# Antimicrobial Properties of CuO Particles Deposited on a Medical Mask

**DOI:** 10.3390/ma15227896

**Published:** 2022-11-08

**Authors:** Agnė Giedraitienė, Modestas Ruzauskas, Rita Šiugždinienė, Simona Tučkutė, Darius Milcius

**Affiliations:** 1 Institute of Microbiology and Virology, Faculty of Veterinary Medicine, Lithuanian University of Health Sciences, Mickeviciaus 9, 44307 Kaunas, Lithuania; 2 Center for Hydrogen Energy Technologies, Lithuanian Energy Institute, 44403 Kaunas, Lithuania

**Keywords:** medical masks, alternatives, CuO, pathogens, coatings

## Abstract

Medical face masks help to reduce the transmission of pathogens, however, the number of infections caused by antimicrobial-resistant pathogens continues to increase. The aim of this study was to investigate the antimicrobial effect of an experimental medical mask layer coated with copper oxide using an environmentally friendly non-thermal physical vapour deposition approach. Pure CuO nanoparticles were successfully deposited on the middle layer of a face mask. The particles were distributed in different size clusters (starting from less than 100 nm dots going up to about 1 µm cluster-like structures). The CuO clusters did not form uniform films, which could negatively influence airflow during use of the mask. We investigated the antimicrobial properties of the experimental mask layer coated with CuO NPs using 17 clinical and zoonotic strains of gram-negative, gram-positive, spore-forming bacteria and yeasts, during direct and indirect contact with the mask surface. The effectiveness of the coated mask layer depended on the deposition duration of CuO. The optimal time for deposition was 30 min, which ensured a bactericidal effect for both gram-positive and gram-negative bacteria, including antimicrobial-resistant strains, using 150 W power. The CuO NPs had little or no effect on *Candida* spp. yeasts.

## 1. Introduction

Continuing COVID-19 pandemics and a reducing supply of new antibiotics mean that scientists have to rethink alternative measures to help against antimicrobial resistance, which is likely to have caused a third more deaths than COVID-19 in 2020 [1]. The development of new antimicrobials is currently avoided due to scientific, regulatory and financial issues [2]. As the number of bacteria resistant to conventional antibiotics grows, alternatives are being investigated, including antibodies, probiotics, bacteriophages and antimicrobial peptides [3]. Prophylaxis, such as wearing masks, as highlighted in the WHO 2022 guidelines, is one of the key measures used to reduce the transmission of the COVID-19 virus [4]. Medical (surgical) face masks are a type of personal protective equipment used to prevent the spread of respiratory infections caused by viruses and bacteria. Masks are trouble-free, easily available, low-priced and clearly efficient [5]. 

Although medical masks are recognised as an effective measure against the transmission of infectious agents, their efficiency is not an absolute [6,7]. The middle filter layer of the three mask layers is the most important, as it protects from particles or droplets carrying viruses and bacteria [8]. The antimicrobial treatment of medical masks was previously explored in order to increase their efficacy [8]. Such treatment could enable the reuse of face masks and at the same time reduce the potential for disease transmission [9]. Antimicrobial systems have already been investigated, including nanoparticles of metal oxides, graphene-based materials, salt compounds (N-halamine-based quaternary ammonium compounds), and different naturally-derived antimicrobial agents [9,10]. Potential systems include nanoparticles of metal, graphene oxides [11] and plant extracts [12].

Nanoparticles of metal oxides act on the potential of the cell membrane in binding the cell walls and releasing metal ions. Such interactions can disrupt the membrane of bacteria and increase oxidative stress, which can damage bacterial proteins [13]. Liquid is thus released from the hyaloplasm [14]. Metal oxides are also characterised by their ability to anchor the wall and further release cationic ions into the solution [15]. As the concentration increases, such cationic ions have a high affinity for the functional groups of the bacterial cell wall, thus disrupting their biological functions and causing the death of the microorganism [13]. The multiple action of metal oxides is potentially a good alternative to antimicrobials [16], which often are ineffective, particularly for the pathogens of nosocomial infections caused by multi-resistant strains of *Staphylococcus*, *Enterococcus*, *Klebsiella*, *Enterobacter*, *Escherichia*, *Acinetobacter* and *Pseudomonas* [17,18].

There are various transition metal oxides such as Ag_2_O [19,20], CuO [21,22], Fe_2_O_3_ [23,24], TiO_2_ [25,26] or ZnO [27,28], which have relatively strong antimicrobial properties characterised by idiosyncratic bacteriostatic mechanisms. Among others, CuO is recognised as a good choice due to its combination of antibacterial efficiency, chemical stability, being a cost-effective material, and because CuO nanoparticles (NPs) do not cause side effects or skin sensitization [29]. CuO is a II-VI group element with good semiconducting property—p-type conductivity with a direct band gap of about 1.74 eV at room temperature. CuO produces reactive oxygen species (ROS) during bacteria or virus inactivation. In bactericidal and bacteriostatic pathways, CuO involves metallic ions and copper-containing materials, inhibiting contaminants by causing oxidative stress, resulting in membrane damage and disrupting protein binding [30]. Alagarasan and co-authors demonstrated that cotton fabrics impregnated with CuO NPs demonstrated a bacterial reduction of more than 90%, which was sustainable even after 20 washing cycles. Various bacteria, namely *Staphylococcus aureus*, *Escherichia coli*, *Pseudomonas fluorescens* and *Bacillus subtilis*, as well as *Candida albicans,* were used during their experiments [29]. Roman and co-authors synthesised CuO NPs onto cotton textiles using the exhaust-dyeing method [30]. It was reported that this structure resulted in between 89.7 and 99.7% bacterial reduction against *Escherichia coli*. Abulikemu et al. demonstrated a more than 99.55% deactivation of human coronavirus 229 E in 30 min with commercially available CuO NP suspensions, confirming the particles’ efficiency as a fast antiviral material [31].

There are some techniques, generally chemical-based methods, for impregnating CuO nanoparticles onto fabrics including the sonication method [32], chemical precipitation [33], the exhaust-dyeing method [34], and others [29,35,36,37]. Chemical-based methods, however, normally involve the following: (i) several steps, which could prolong the total synthesis time; (ii) using various additional materials or solutions, which could affect the emergence of impurities in the synthesised material; and (iii) environmental issues, as various solid or liquid wastes can be produced during material synthesis.

This research used the non-thermal physical vapour deposition (PVD) technique in order to overcome the above issues. PVD is recognised as a versatile, one-step, environmentally friendly process, in which the synthesised materials are characterised by high purity and good adherence on substrate material [38,39]. To our current knowledge, no article has reported the use of this technique for CuO NP deposition on a middle filter layer in medical masks.

The aim of this study was to investigate the antimicrobial effect of an experimental medical mask layer coated with copper oxide using an environmentally friendly non-thermal physical vapour deposition approach.

## 2. Materials and Methods

### 2.1. Coating of the Middle Mask Layer and Glass Slide with CuO Nanoparticles

**Figure 1:**
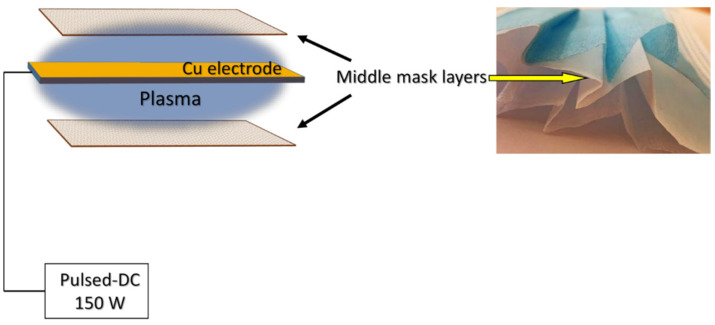
Experimental set up of CuO synthesis on the middle mask layer.

CuO nanoparticles were deposited on the surface of the middle mask layer (dimensions: 12 cm × 15 cm) using a physical vapour deposition system. The middle layer was chosen to minimize direct contact of CuO with human skin and limit the possibility of inhaling particles due to the known toxicity of CuO. Two samples of the mask layers were placed in a vacuum chamber with a Cu electrode between them during deposition (Figure 1). A pulsed-DC power source (P = 150 W) was used for plasma generation. During the deposition process, oxygen was supplied into the vacuum chamber to maintain a constant pressure of 40 Pa for the CuO NPs. The distance between the Cu electrode (dimensions 12 cm × 15 cm; 99.99% purity) and the sample was 5 cm. CuO NPs were deposited for 15, 30, 60 and 120 min. The mask material was not favourable for the direct observation of bacterial grown, so glass slides were also used as the substrate for a better microscope resolution.

### 2.2. Chemical and Structural Characterisation of the Deposited CuO Nanoparticles

Surface views of CuO-coated mask fabric and bacteria grown on the glass substrates were investigated by scanning electron microscope (SEM, Hitachi S-3400 N, Tokyo, Japan) using a secondary electron detector. Elemental mapping of the middle mask layers with CuO nanoparticles was performed using energy-dispersive X-ray spectroscopy (EDS, Bruker Quad 5040, Hamburg, Germany). The crystal phase of the CuO was identified by an X-ray diffractometer (XRD, Bruker D8, Hamburg, Germany) operating with Cu Kα radiation in the 2θ range between 20° and 70°.

### 2.3. Strains of Microorganisms

Seventeen reference, clinical and zoonotic strains of gram-negative and gram-positive bacteria and yeasts were used. The susceptibility testing of microorganisms, previously isolated at the Microbiology and Virology Institute of the Lithuanian University of Health Sciences, was performed according to the EUCAST guidelines [40]. The strains had the following resistances to antibiotics: *Enterobacter cloaceae* (*E. cloacae*) (resistance: ampicillin, sulfamethoxazole/trimethoprim, gentamicin, cefoxitin), *Klebsiella pneumoniae* (*K. pneumoniae*) (resistance: ampicillin), *Salmonella enterica* (*S. enterica*) (resistance: none), *Citrobacter freundii* (*C. freundii*) (resistance: ampicillin, ciprofloxacin, cefoxitin, amoxicillin/clavulanic acid), *Pasteurella multocida* (*P. multocida*) (resistance: ampicillin; SXT—sulfamethoxazole/trimethoprim, tetracycline, ampicillin, ciprofloxacin), *Acinetobacter baumanii* (*A. baumanii*) (resistance: gentamicin, ciprofloxaicin, amikacin, imipenem, meropenem), *Staphylococcus haemolyticus* (*S. haemolyticus*) (resistance: penicillin, erythromycin, cefoxitin, ciprofloxacin), *Enterococcus faecium* (*E. faecium*) (resistance: penicillin, tetracycline, quinupristin/dalfopristin) and *Candida tropicalis* (*C. tropicalis*) (antifungal resistance: amphotericin B, ketokonazole, miconazole, fluconazole). The ATCC strains tested included *Escherichia coli* ATCC 25922, *Proteus mirabilis* ATCC 25933, *Pseudomonas aeruginosa* ATCC 27853, *Aeromonas hydrophila* DSM 112649, *Staphylococcus aureus* ATCC 25923, *Bacillus cereus* ATCC 11778, *Enterococcus faecalis* ATCC 29212 and *Candida albicans* ATCC 10231.

### 2.4. Assessment of Antimicrobial Activity

**Figure 2:**
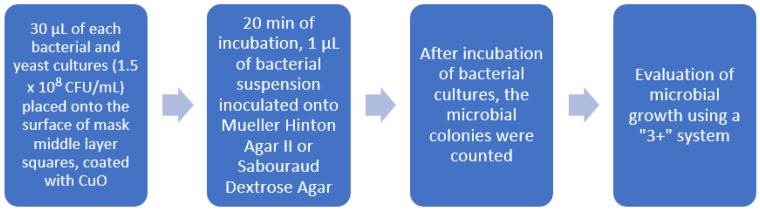
Preparation of microorganisms for the evaluation after direct contact with CuO nanoparticles.

The experimental mask’s middle layer was coated with CuO NPs for deposition times of 15 min, 30 min, 60 min and 120 min. The material was cut into squares of 10 mm × 10 mm using sterile scissors and then placed into an empty sterile Petri dish. Before the experiment, the cut squares also were tested for sterility using thioglycollate medium (CM0173, Thermo Fisher, Scientific, Basingstoke, UK). Bacteria and yeast cultures were diluted with sterile saline up to a 0.5 McFarland Unit density (~1.5 × 10^8^ CFU/mL), and 30 µL of each culture was placed onto the surface of ready-prepared material squares coated with CuO. The suspension was spread evenly using a plastic bacteriological loop. After 20 min of incubation at room temperature, the sample for inoculation (1 µL) was taken from the surface of the mask layer squares using a sterile plastic bacteriological loop (1 µL). The sample was then inoculated onto either Mueller Hinton Agar II (Thermo Fisher, city, UK) or Sabouraud Dextrose Agar (for fungi, Thermo Fisher, Basingstoke, UK). Incubation time was up to 48 h at +35 °C for each culture, except for the yeasts and *Aeromonas hydrophila*, +25 °C, with the researchers constantly checking on the microbial growth of the cultures. After incubation of bacterial cultures, the growth was evaluated by counting the number of bacterial colonies. The intensity of the growth was evaluated according to the “3+” system (Table 1). In the absence of growth, the intensity was evaluated as “0”, growth from 1 to 10 colonies (10^6^–10^7^ L)—“+”, 11 to 100 colonies (10^7^–10^8^ L)—“++”, and ˃100 colonies (˃10^8^ L)—“+++”. The same experiment was repeated three times. The course of the experiment is presented in Figure 2.

For control purposes, the middle layer of a 3-ply medical mask (XianTao Hong Tai Health & Safety Protective Co., Ltd., Xiantao, China) was used and tested in the same way as the experimental mask layer with CuO.

**Figure 3:**
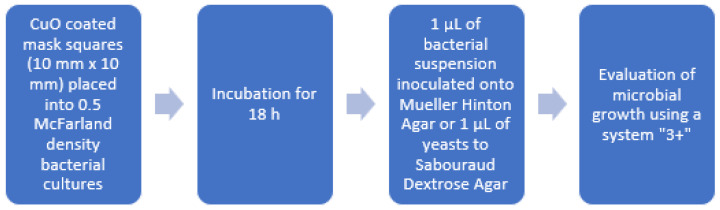
Preparation of microbes for evaluation after indirect contact with CuO nanoparticles.

The second part of the experiment was performed to investigate whether the microorganisms could survive in close but not direct contact with CuO-coated mask material. Suspensions of microorganisms of 0.5-McFarland density (~1.5 × 10^8^ CFU/mL) were prepared, and CuO-coated mask squares (1 cm × 1 cm) were placed into the tubes (containing 2 mL of the suspension), mixed using tube mixer for 5 s, and placed into a thermostat at +35 °C overnight. A total of 1 µL of bacterial suspension was inoculated onto Mueller Hinton Agar (Thermo Fisher, Basingstoke, UK) and incubated at +35 °C for 48 h (+25 °C for yeasts and *A. hydrophila*). The colonies on the agar surface were counted. The growth was scored using the “3+” system according to the number of colonies. The course of the experiment is presented in Figure 3.

### 2.5. Visual Evaluation of the Direct Contact of Microorganisms with CuO Nanoparticles

Bacterial suspensions of 1 µL of 0.5-McFarland-unit-density gram-positive (*S. aureus*), gram-negative (*E. coli*) and spore-forming bacteria (*B. cereus*) were transferred onto CuO-coated glass slides (deposition time 120 min) and kept at ambient temperature for 5 min until the suspension dried. Then, the smears were fixed using 2.5% (*w*/*v*) glutaraldehyde in 0.05 M sodium cacodylate buffer (Sigma-Aldrich, Burlington, MA, USA) at 4 °C for 2 h followed by fixing with 1% of osmium tetroxide in cacodylate buffer (Sigma-Aldrich, Burlington, MA, USA) for 60 min at 4 °C. The fixed samples were prepared for electron microscopy by dehydration using ethanol solutions of 25% (*v*/*v*), 50%, 70%, 95% and 100% for 10 min each. The same smears were performed on glass slides without coating for control purposes.

### 2.6. Statistical Analysis

Statistical analysis was performed using the R statistical package, version 3.6.2 (R-project.org; accessed on 1 September 2022). Results were considered statistically significant when *p* < 0.05.

## 3. Results

### 3.1. Structural Analysis

**Figure 4:**
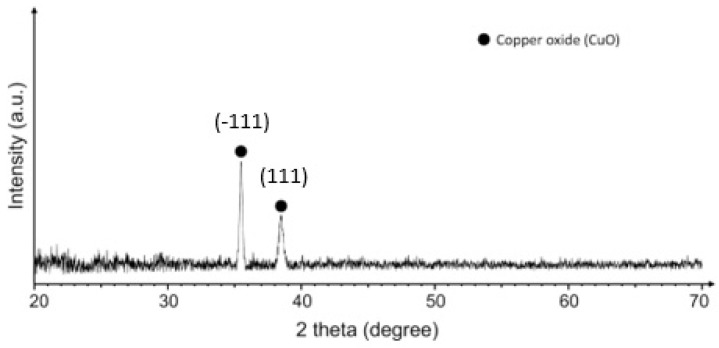
XRD pattern of CuO nanoparticles deposited on quartz at 150 W for 120 min.

XRD analysis was performed to confirm the crystalline structure of the as-deposited CuO NPs. The surface of the mask layer was inconvenient for the direct XRD measurements, therefore, the CuO nanoparticles were deposited on a flat quartz substrate under the same conditions and the XRD data were collected from the flat quartz sample (Figure 4). The XRD pattern correlated well with the monoclinic copper (II) oxide structure (CuO, JCPDS card number 04-015-5869). The characteristic diffraction peaks of CuO at 2Θ = 35.49° and 38.48° corresponded to the (−111) and (111) crystal planes, respectively.

The size of the crystallites was calculated using the Scherrer equation and it was found that the average size of the crystallites was 31 nm, which corresponded to nano-scale crystallites.

### 3.2. The Measurements of Elemental Mapping and Concentration

**Figure 5:**
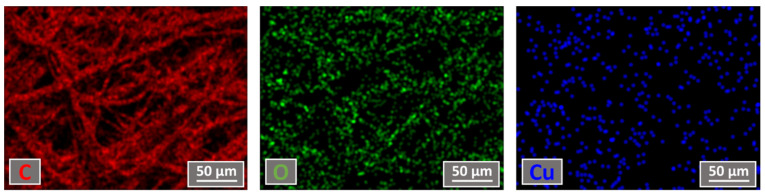
Elemental mapping views of samples deposited for 120 min.

The elemental mapping and concentration were measured using the EDS technique and the results obtained after 120 min of deposition are presented in Figure 5. An elemental composition measurement revealed that the middle layer of the mask consisted of around 89 at.% carbon, 10 at.% oxygen and up to 1 at.% of copper after CuO deposition. This indicated a relatively slow CuO deposition process using 150 W power. Our primary experiments also showed that only a small increment in deposition power led to the middle layer of the mask overheating and the deposited structure starting to crack. A power rating of 150 W for the CuO deposition process was therefore selected.

**Figure 6:**
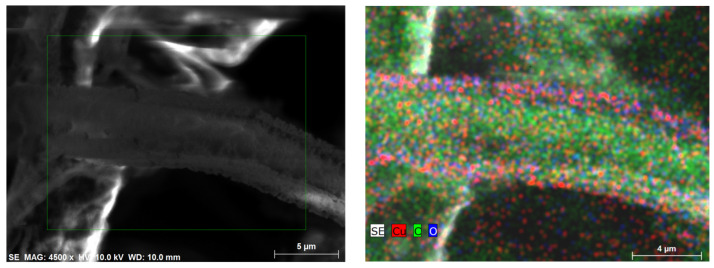
Cu particle distribution on mask surface. SEM images of the middle mask layer on the left. EDS elemental mapping showing Cu particles distribution on the right.

Elemental mapping was performed in order to understand the Cu particles’ distribution on the surface of the middle layer of the mask. The results showed that Cu was distributed relatively uniformly over the whole surface of the mask, avoiding particle agglomeration and the formation of uniform, continuous thin films, which can negatively impact the mask pore size by covering it with CuO film. A more precise analysis of the Cu particle distribution on the mask material showed (Figure 6) that the Cu particles were distributed in the form of different clusters. The size of the clusters varied a lot, starting from less than 100 nm dots going up to about 1 µm cluster-like structures.

This result confirmed that the CuO was uniformly deposited on mask material surface and did not cover the pores with a uniform film, which could prevent air flow during the use of the mask.

### 3.3. Antimicrobial Activity of Coated Material

**Figure 7:**
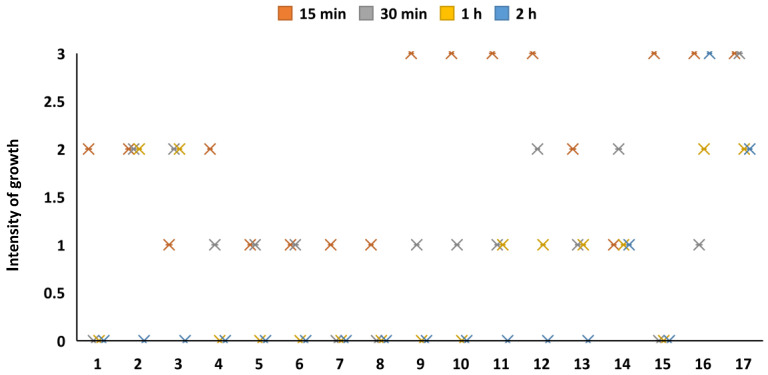
Antimicrobial potential of Cu oxide coatings with direct inoculation onto the mask layer. 1—*E. cloacae*, 2—*E. coli*, 3—*K. pneumoniae*, 4—*P. mirabilis*, 5—*S. enterica*, 6—*C. freundii*, 7—*A. hydrophila*, 8—*A. baumannii*, 9—*P. aeruginosa*, 10—*P. multocida*, 11—*S. aureus*, 12—*S. haemolyticus*, 13—*E. faecalis*, 14—*E. faecium*, 15—*B. cereus*, 16—*C. albicans*, 17—*C. tropicalis*.

All of the bacterial strains were able to grow on the nutrient media after direct inoculation of the cultures taken from the control, i.e., the medical mask surface containing no antimicrobial materials. The antimicrobial activity of the CuO on the bacteria depended on the deposition time of the CuO (15 min, 30 min, 60 min and 120 min) on the mask layer. All the microbial cultures survived with a deposition time of 15 min during the direct contact experiment, however, different species grew unequally. The growth of some cultures was evaluated as 3+, whereas that of others was evaluated as “++” or “+” (Figure 7). There were no differences in the growth rates between gram-positive and gram-negative bacteria. The bactericidal effect was much more powerful with an exposure of 30 min; only a single microorganism (*Candida tropicalis*) grew as “3+”, whereas four bacterial strains were fully inactivated, and the rest were partially inactivated (“+” or “++”). A longer exposure time had a better bactericidal effect; nine strains of microorganisms were fully inactivated within a 60 min exposure, whereas 14 out of 17 strains were fully inactivated with a deposition of 120 min. The bactericidal effect was therefore directly dependent on the duration of CuO deposition on the mask layer (*p* < 0.05). The experiment demonstrated that the yeasts (*C. albicans* and *C. tropicalis*) were not affected by the CuO nanoparticles, and those strains had the same ability to grow after direct exposure to CuO as on the control mask layer without metal oxide. Only a single bacterial species (*E. faecium*) was able to survive (“+”) after direct contact with the CuO during the longest deposition (120 min).

**Figure 8:**
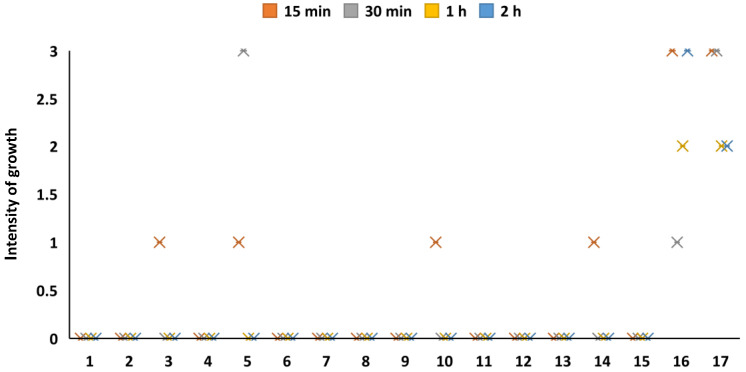
Antimicrobial potential of CuO coatings with indirect exposure to microorganisms. 1—*E. cloacae*, 2—*E. coli*, 3—*K. pneumoniae*, 4—*P. mirabilis*, 5—*S. enterica*, 6—*C. freundii*, 7—*A. hydrophila*, 8—*A. baumannii*, 9—*P. aeruginosa*, 10—*P. multocida*, 11—*S. aureus*, 12—*S. haemolyticus*, 13—*E. faecalis*, 14—*E. faecium*, 15—*B. cereus*, 16—*C. albicans*, 17—*C. tropicalis*.

Figure 8 demonstrates the growth ability of the microorganisms after a prolonged period with indirect contact with the experimental mask layer, i.e., when the layer square was placed into a bacterial suspension. The results demonstrated that the mask layer coated with CuO had an excellent bactericidal effect on all the tested cultures, except for *S. enterica*, when the CuO deposition time was not less than 30 min. *S. enterica* was inactivated only when the CuO exposition time was at least 120 min. A deposition time of 15 min was effective on all the test cultures, except for *K. pneumoniae, S. enterica*, *P. multocide, E. faecium* and the yeasts. Overall, indirect exposure to the CuO-coated mask material had a very low effect on the yeasts, and they were able to survive after the longest deposition (120 min) of CuO on the mask layer.

**Figure 9:**
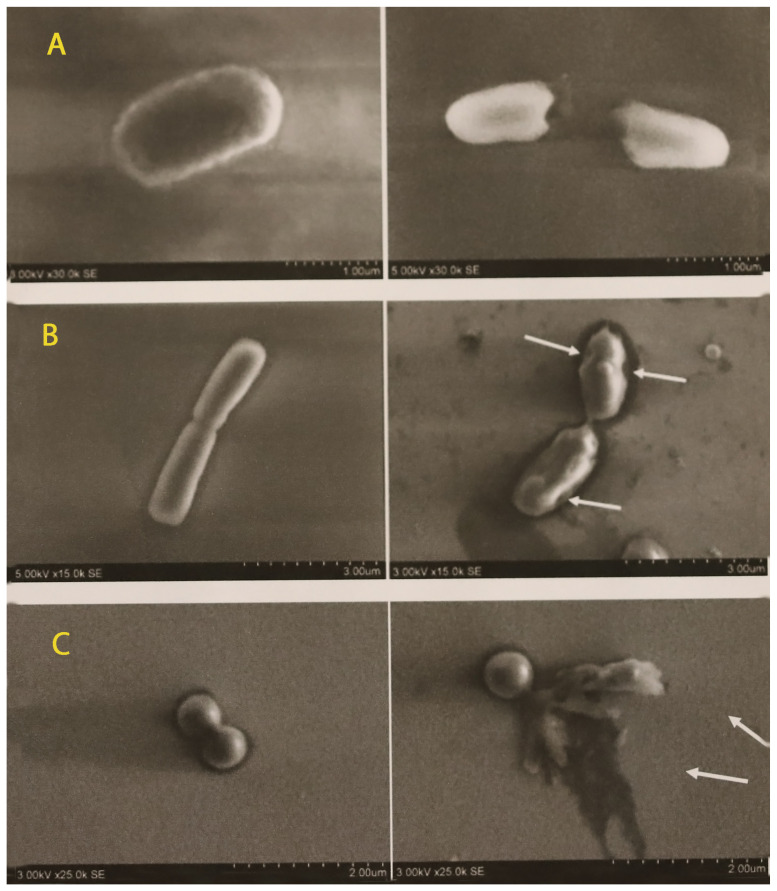
SEM micrographs with non-treated (**left**) and copper-treated (**right**) bacterial images: (**A**)—*Escherichia coli* ATCC 25922; (**B**)—*Bacillus cereus* ATCC 11778; (**C**)—*Staphylococcus aureus* ATCC 25923. Damaged cells are marked by arrows.

Figure 9 shows SEM micrograph images after the bacterial cultures were transferred onto a glass slide coated with CuO. The damaged bacterial cells are visible in the pictures after contact with the CuO NPs.

## 4. Discussion

The results of this study demonstrated that CuO nanoparticles coated on medical mask layer could be effective against multiple pathogens. Bacterial inactivation, however, depended on the deposition time of the nanoparticles on the mask layer. The optimal bactericidal action was reached when the mask layer was coated with a CuO deposition time of 120 min. In this case, a short contact time with bacteria (20 min on the mask material) was enough to kill them. The SEM micrographs demonstrated that some bacteria, particularly the gram-negative *E. coli* cells, were disrupted even after 5 min (during drying of the bacterial suspension on the glass surface) direct contact with the coating. There was also a good bactericidal effect with a smaller amount of CuO (deposition time from 30 min to 60 min) with more prolonged indirect contact (24 h) when the coated mask layer was placed in the bacterial suspension.

Recent studies have demonstrated that CuO NPs have an antimicrobial effect on *E. coli*, *S. aureus*, *Bacillus subtilis and P. aeruginosa* [41], but there is a lack of information about its antimicrobial effects on a wider spectrum of bacterial or fungal species. Our results demonstrated very low or no antifungal effect on the yeasts, which supported data obtained by other authors [42].

The surface layer of a surgical mask is hydrophobic and dry. Protection against microorganisms could be limited if the mask becomes wet with the wearer’s body fluids or respiratory droplets. Microorganisms can then penetrate this layer’s surface [43]. It is difficult to predict the contact of bacteria with the mask middle layer in field conditions as it may depend on the size of bacteria, breathing intensity and the density of mask layers. It should be assumed that antimicrobial action has to be fast, and therefore a higher concentration of Cu oxide, which has a faster effect in a shorter time, should be considered for future applications. High concentrations of metal ions on the mask surface could lead to high exposure for the mask users however, as the copper oxide particles may be released from the surface and reach the respiratory system. For this reason, the next step should be evaluating the stability of Cu oxides on the mask layer, and this should include a safety risk assessment. Long studies have indicated that oral copper exposures are typically not a human health concern [44]. Copper is an essential microelement required by adults in amounts of 1 to 100 mg/day and it is found in high concentrations in the brain, liver and kidneys [45]. Overexposing doses of copper can induce toxicity symptoms, however, and intoxication by copper usually occurs through contaminated food and water sources. A study performed by Lai et al. demonstrated that CuO NPs can induce pulmonary fibrosis in mice [46]. In vitro studies have demonstrated that CuO NPs induce cytotoxic, genotoxic and oxidative stress responses in several cultured human lung epithelial cells and that the toxicity level is dose-dependent [47,48]. In vivo studies on the lung toxicity of CuO NPs are largely lacking [48]. Karlsson et al. showed that CuO NPs were much more toxic than CuO micrometer particles [49]. Different technologies have been investigated in order to increase the safety of nanoparticles. For example, the toxicity of CuO NPs to different cell lines was decreased when Fe-doped CuO NPs were used [50]. Special technologies are used to increase the binding of NPs on textiles, such as the one-pot modification technique (pad–dry–cure) using carboxymethyl chitosan as a binder and stabiliser [51]. Chitosan can be used to improve the adhesion of metal salts or NPs on cotton, linen, polyamide and aramid fabrics [52] in order to reduce exposure to the respiratory system. The biosynthesis of NPs using plant extracts is also promising as it enables biocompatible and water-soluble NPs with good stability and improved antimicrobial and antioxidant properties to be obtained [53,54,55]. The green nanobiotechnological synthesis of NPs using biomolecules (proteins, enzymes, DNA and plant extracts) has become a rapidly developing research area. Green synthesis methods have overcome the disadvantages of traditional physical and chemical synthesis approaches, such as high cost, long time scales and toxicity [56,57].

We did not detect the clear dependence of the Cu oxide action on the type of bacteria in this experiment. The gram-positive, gram-negative and spore-forming bacteria remained alive in shortest duration of Cu oxide exposure, but all were inactivated when the mask layer was coated with a higher concentration (higher deposition) of CuO. The SEM micrographs demonstrated damage to the gram-negative bacteria (*E. coli*) and lesser damage to the gram-positive ones. The experiments proved that both gram-negative and gram-positive bacteria, including spore-forming bacteria, were fully inactivated depending on the deposition time of CuO. During the experiment with direct contact with the mask layer, only a single species of bacteria—*Enterococcus faecium*—was able to survive after contact with the layer coated with the highest concentration of CuO. There might be a few reasons for its resistance to CuO, including intrinsic species resistance or acquired strain resistance (co-resistance or genes encoding resistance to heavy metals), and further experiments in this direction could help to answer this question. Studies performed by other authors have demonstrated that CuO particles are effective against both gram-positive and gram-negative bacteria, however, the effectiveness of CuO depends on the size of the particles. In a study performed by Azam et al., smaller particles demonstrated a higher activity, whereas the minimum inhibitory concentration of CuO NPs annealed at 400 °C was the lowest for all the tested bacterial strains [21]. The data suggests that smaller copper oxide particles are more effective but also more toxic. This correlation should be considered in the different applications of copper oxide coatings.

The inactivation mechanisms of bacteria by metal ions are different. As a typical example, bare CuO NPs with a positive charge at neutral pH can effectively adhere to the negatively charged bacterial cell wall via electrostatic interactions and inhibit the physiological functions necessary for cell metabolism [13]. The bactericidal mechanism of metal and metal oxide can also be associated with the production of reactive oxygen species, which includes superoxide radical anions, hydrogen peroxide anions and hydrogen peroxide, which interact with the cell walls of bacteria causing damage to the cell membrane and in turn inhibiting the further growth of cells with the leakage of internal cellular components, leading to the death of bacteria [58]. The rupture of bacterial cell walls and leakage of cytoplasm, particularly in *Escherichia coli* as well as in some cells of *Staphylococcus aureus,* was visible in the micrographs taken in this study. This data supported previous findings that CuO acts against a wide range of bacteria by disrupting cell walls and distorting helical DNA structures [59].

## 5. Conclusions

Pure CuO nanoparticles were successfully deposited using an environmentally friendly non-thermal physical vapour deposition approach. The particles were distributed in different size clusters (starting from less than 100 nm dots going up to about 1 µm cluster-like structures). The CuO clusters did not form uniform films, which could negatively influence airflow during use of the mask.

The antimicrobial effect depended on the deposition time of the CuO NPs on the medical mask layer. The CuO NPs demonstrated a strong antibacterial effect on gram-negative, gram-positive and spore-forming bacteria, including antimicrobial-resistant and wild bacterial isolates, when the deposition time using 150 W power was not less than 30 min. The CuO NPs had little or no effect on *Candida* spp., independent of the duration of NP deposition. The SEM images demonstrated the disruption of cell membranes and cell lysis in the bacteria after their contact with CuO NPs.

## Figures and Tables

**Table 1:** Evaluation of microbial growth based on their ability to form colonies after exposure to CuO nanoparticles.

Intensity of Growth Measured by the Number of Bacterial Colonies	Growth Level Using “3+” System
No growth	0
1 to 10	+ or 1
11 to 100	++ or 2
˃100	+++ or 3

## Data Availability

The data presented in this study are available on request from the corresponding author. The data are not publicly available, due to the next work.
